# Beyond Algorithmic Oversight: Internal Morality of Medicine and Meaningful Human Control in AI-Assisted Care

**DOI:** 10.3390/healthcare14121638

**Published:** 2026-06-10

**Authors:** Aleksej Omeljančiuk, Eimantas Peičius, Aušra Urbonienė, Gvidas Urbonas

**Affiliations:** Department of Bioethics, Lithuanian University of Health Sciences, LT-44307 Kaunas, Lithuania; eimantas.peicius@lsmu.lt (E.P.); ausra.urboniene@lsmu.lt (A.U.); gvidas.urbonas@lsmu.lt (G.U.)

**Keywords:** artificial intelligence, medical ethics, meaningful human control, clinician–patient relationship, clinical decision-making

## Abstract

**Background/Objectives**: Artificial intelligence reshapes clinical practice, and its effect on the clinician–patient relationship requires reconsideration of the frameworks that have shaped modern medical ethics. When clinicians delegate expertise to algorithms they cannot verify, it becomes unclear who bears clinical responsibility. **Methods**: This article applies a theoretically grounded normative approach to explore the ethical conditions under which artificial intelligence can be integrated into clinical practice without compromising the moral foundations of medicine. The analysis is primarily based on Pellegrino and Thomasma’s concept of the internal morality of medicine and the clinician’s act of profession. It further draws on Kantian ethics of human dignity, Levinasian relational ethics, virtue ethics, and Vallor’s concept of technomoral wisdom. **Results**: AI systems do not satisfy the conditions under which moral responsibility can be ascribed to them. Clinical moral agency lies in the capacity to bear three distinct responsibilities—epistemic, relational, and phronetic—none of which can be fulfilled by AI. The implementation of AI in healthcare, therefore, must occur strictly under the condition of Meaningful Human Control, rather than as a technical function of human oversight over algorithmic outputs. To ensure that MHC can function as an effective and ethically grounded safeguard, we propose five normative requirements: primacy of clinical judgement, prohibition of forced automation, traceability and explainability, transparency towards patients, and retaining clinical authority. Dialogue between clinicians and patients should remain the foundation of clinical decision-making. The proposed normative requirements aim to preserve the internal morality of medicine in a form that harmoniously combines both technological progress and established medical ethics.

## 1. Introduction

Among the many ways artificial intelligence (AI) is reshaping clinical practice, its effect on the clinician–patient relationship is particularly significant. Parsons [1] noticed that the clinician–patient relationship is asymmetric, whereby the patient becomes dependent by assuming the role of the sick. Patients rely on someone who has better knowledge and skills to tackle health issues. As an alternative to the paternalistic approach, new models started emerging together with the theoretical framework elaborated by Szasz and Hollender [2]. These models tackle epistemic and power asymmetry through recognition of and respect for the patient’s autonomy, dignity, and rights.

Subsequently, the principles of trust, autonomy, and shared decision-making were incorporated into international guidelines and became an integral part of the professional standard. However, the rapid integration of AI into clinical practice introduces new forms of epistemic asymmetry, as both clinicians’ and patients’ knowledge become increasingly dependent on systems with a nearly limitless knowledge base [3,4].

The benefits of AI integration in healthcare are well established and extensively documented elsewhere—ranging from improved diagnostic accuracy and patient care to substantial reductions in healthcare professionals’ workload [5,6,7]. Rather than revisiting these widely acknowledged advantages, this paper focuses on the ethical tensions that arise alongside them, particularly those concerning clinician accountability, transparency, and professional autonomy. Clinicians are increasingly held responsible for the outputs of algorithms whose limited transparency may generate confusion, anxiety, and persistent uncertainty [8,9,10], while overreliance on AI risks eroding clinical judgement [6]. This tension calls for a deeper examination of what it means to practise medicine amid shifting roles and responsibilities, particularly when (semi-)automated systems recommend high-stakes decisions while the ultimate burden of liability remains with humans.

Before the AI era, Pellegrino and Thomasma [11] defined medicine fundamentally as a moral enterprise aimed at the good of the ill person, and this is precisely what determines the internal morality of medicine. According to Pellegrino, “medicine is a moral community because its practitioners are bound together by a common goal—the good of the patient” [12]. Medical practice serves as a response to the patient’s call for help, and clinicians take a moral commitment to treat the patient and reduce suffering [13]. In this context, there is a doubt as to whether the integration of AI into healthcare systems will be able—and, if so, under what conditions it will be able—to sustain the moral goal of medicine. AI systems can calculate the probability of a diagnosis or the potential success of treatment options, but they cannot determine what the right clinical judgement would be. Clinical rightness requires synthesising empirical data with the patient’s values and lifeworld and inherently relies on value-based judgements. Delegating such decisions to AI may increase the risk of eroding the clinical relationship (which respectively gives medicine its moral authority) and may threaten professional autonomy to make independent, carefully deliberated choices by overreliance—either voluntary or involuntary—on those assistive (AI) algorithms.

We argue that AI systems cannot perform what Pellegrino calls ‘the act of profession’—the public commitment to care for the sick with competence and virtue. As a result, integrating such AI systems into clinical practice creates gaps in accountability that arise from the opacity of algorithms and their uncritical implementation.

Neither the system nor the clinician can fully calculate the decisions the system has shaped. When clinicians delegate expertise to opaque algorithms they cannot independently verify, it becomes unclear who bears clinical responsibility: the clinician using the system or the developers who built it. This ambiguity creates the responsibility gap that may lead to accountability disparities [14].

In this paper, we claim that AI integration into healthcare—by enhancing diagnostic accuracy, enabling personalised treatment, supporting clinical decision-making, optimising population health management, and reducing both costs and the workload burden on healthcare professionals—should be accompanied by the clinician’s professional responsibility and moral commitments to the patient [15,16]. AI systems, particularly clinical decision-support tools, are designed to extend, complement, and improve clinician judgement rather than replace it [17]. Such AI-assistance can return time and attention to the patient [8], thereby widening the space for the relational and interpretive dimension of the encounter rather than narrowing it. What we explore in this article are the conditions under which this extension preserves rather than displaces the clinician’s deliberative space needed to retain responsibility for recognising and overriding outputs that do not fit the patient’s situation.

However, clinical interpretation of AI outputs does not take a single form. The thing to be interpreted differs across AI modalities; therefore, the interpreter’s task may take a different shape across them. “AI in healthcare” is not a single technology but a family of systems that mediate clinical judgement in different ways. Diagnostic image-analysis tools recognise patterns in radiological, dermatological, or retinal data; clinical decision-support systems (CDSS) stratify risk and propose treatment options by integrating heterogeneous patient data; ambient and generative tools transcribe, summarise, and increasingly structure the clinical encounter itself. Each modality places the clinician in a distinct relation to the system.

Because the moral responsibility and deliberative essence of clinical practice cannot be delegated to machines, our central argument is that the integration of AI into healthcare should therefore be managed under ‘Meaningful Human Control’ (MHC) [18] as a positive condition for the clinician’s exercise of phronesis as ‘technomoral wisdom’ [19]. In technological environments, clinicians are expected not only to operate the tools but also to assume the role of a moral interpreter in order to assist patients in seeking decisions that best meet their values and life circumstances. This deliberative communication between the clinician and the patient has long served as a normative ideal in medicine. However, for this purpose, healthcare professionals should be given full professional autonomy to exercise decisional authority over algorithmic outputs in order to prevent the treatment process from turning into a purely technological one [20,21]. In this context, Meaningful Human Control must be assured in deliberative communication if we want to maintain the autonomy and dignity of both clinicians and patients. Although the framework we present here is normative, it provides a necessary theoretical justification for the need to sustain the human factor at all levels of AI-mediated clinical encounters.

## 2. Methods

### 2.1. Study Design

This article applies a theory-driven normative approach to examine the ethical conditions under which artificial intelligence may be integrated into clinical practice while preserving the moral foundations of medicine. More precisely, this study constitutes a normative bioethical analysis informed by a targeted, non-systematic literature review. The methodological approach is conceptual and interpretive, aiming to thematically synthesise existing research and develop new normative insights, rather than to aggregate empirical findings statistically.

### 2.2. Theoretical Framework and Its Justification

The analysis is grounded primarily in Pellegrino and Thomasma’s [11] interpretation of the internal morality of medicine and the clinician’s act of profession. This conceptual framework was selected because it views medicine as a moral practice oriented toward the good of the patient through the clinician’s phronesis: the virtue-based insight into what is good for this patient. We chose the Pellegrinian approach because it is comparatively resistant to the technologisation of medical practice—a risk that grows with the rise of autonomous or quasi-autonomous AI systems. The analysis further relies on Kantian ethics of human dignity, Levinasian relational ethics, virtue ethics, and Vallor’s [19] concept of technomoral wisdom. These frameworks are used to clarify why clinical judgement cannot be reduced to technical prediction and why moral responsibility in medicine remains tied to human agency, relational presence, and practical wisdom.

In choosing this framework, we tried to find a way between two influential but, for our purposes, incomplete approaches. Principlism was not chosen because any attempt to list clear ethical principles risks getting codified and built into algorithms that may at some point overrule the clinician’s not only clinical, but also ethical judgement instead of informing it. The ethics of care, on the other hand, reminds us that relationship is what makes medicine human, but, in its purely relational form, it is not sufficiently clear how a clinician takes responsibility for a particular patient. By reading Pellegrino and Thomasma’s account, we seek to determine the preconditions that retain a moral space for the clinician to deliberate ethically sensitive cases, regardless of whether they apply a principlist, an ethics-of-care-based, or any other ethical framework in between.

Our argument proceeds in four stages. First, we reconstruct the ethical significance of the clinician–patient relationship in terms of vulnerability, dignity, trust, and clinical judgement. Next, we examine whether AI systems can satisfy the conditions required for moral agency in clinical practice. Then, we analyse how AI-mediated care may affect trust, professional autonomy, and patient dignity. Finally, we derive normative requirements for Meaningful Human Control in AI-assisted healthcare, where clinicians are given deliberative space to act as moral interpreters between the algorithmic output and the singularity of the patient.

### 2.3. Literature Search and Selection Strategy

Besides the fundamental philosophical works of Pellegrino and Thomasma, Kant, and Levinas, the empirical literature on AI implementation in healthcare was used to contextualise the normative analysis. These sources were identified through targeted searches in the PubMed, Scopus, and Google Scholar databases using keywords such as “artificial intelligence,” “dignity,” “Pellegrino,” “clinical decision support,” “automation bias,” “responsibility gap,” and “meaningful human control.” For our analysis, we chose peer-reviewed publications in English. Purely technical papers without ethical or clinical relevance were excluded. The sources were selectively used to ground the normative argument rather than to provide a complete review of the empirical evidence. Regulatory and governance documents, particularly the EU AI Act and the WHO guidance on the ethics and governance of AI for health, were examined as current institutional reference points.

## 3. The Internal Morality of Medicine and the Dimensions of Dignity

Medicine cannot be reduced to applied biology or a transactional service. Drawing on the Aristotelian and Thomistic traditions, Pellegrino argues that medical practice must rely on phronesis—prudent judgement or practical wisdom—which functions as the link between the intellectual virtues, enabling us to know, and the moral virtues, enabling us to act well. It encompasses the ability to identify suitable means in complex situations in order to advance the genuine ends of the healing relationship. As the power of discernment, prudence translates in the clinical context into clinical judgement: the capacity to determine how, when, and in what way to act under conditions of uncertainty, in situations never before encountered, or in cases where the virtues themselves come into tension [22].

Illness, according to Pellegrino [12], is not just a breakdown of the body, but an ‘ontological assault’ that compromises the patient’s freedom, rationality, identity, and self-determination. Therefore, the main goal (telos) of medicine is not purely epistemic, but entails the ‘internal morality’ that is derived from the face-to-face clinical encounter between the clinician and the patient [12]. In this encounter, the clinician faces not a clinical case but a vulnerable person, and it is here that the Kantian moral imperative “to treat human being never merely as a means but always as an end itself” [23] is met. The obligation to consider the patient’s best interests stems from the moral obligation to respect and protect the ‘essential and inviolable core of humanity’ in each person [24]. Even though dignity can be compromised by adverse individual and organisational conditions [25,26,27], healthcare professionals are expected to honour their patients’ esteem and take measures to mitigate their severe dependency [28].

Illness compromises physical and psychological independence. This condition calls for what Pellegrino terms the clinician’s ‘act of profession’. In making such a declaration, the clinician publicly commits to using specialised clinical knowledge exclusively for the patient’s healing and well-being. The clinician–patient relationship is therefore founded upon a bond of trust and moral obligation rather than the mere application of technical solutions. Within this moral framework, Pellegrino argues that comprehensive clinical judgement must answer three consecutive questions. The clinician must first determine ‘what can be wrong’ and ‘what can be done’, and then decide ‘what should be done for the patient’ [13]. Classical diagnostic tools historically helped clinicians discover what could be wrong. Today, AI systems actively assist in addressing ‘what can be done’ by outlining treatment alternatives. Answering the final question of ‘what should be done’ requires phronesis—the practical wisdom to integrate empirical knowledge with the patient’s values and context [29].

This need to address vulnerability demonstrates that safeguarding human dignity cannot be reduced solely to technical parameters or the results of calculations [30]. Genuine care and respect for the patient’s dignity require a relational autonomy approach in which an experienced specialist is able to truly hear, see, and value the patient [31]. Since medicine is a moral practice oriented toward the good of particular persons, honouring this dignity demands the active, empathetic engagement of a moral agent who understands the need to preserve patient-centred care as a whole [32]. It is within the clinician’s moral responsibility to synthesise empirical data with human values and the unique perception of the good, and, on that basis, to take what Pellegrino calls the ‘right action’ [29].

## 4. AI, Moral Agency, and Moral Responsibility

The claim that only the clinician can determine what should be done presupposes that clinical responsibility belongs to a moral agent, and, here, the integration of AI into clinical judgement becomes conceptually problematic. AI systems increasingly shape the evidential basis of medical decisions, yet they do not satisfy the conditions under which moral responsibility can be ascribed. Medical decisions require a genuine capacity for moral choice. Therefore, a clinician’s accountability cannot be transferred to a tool or system that lacks the capacity for ethical judgement. Specifically, clinical judgement lies in the capacity to bear three distinct responsibilities—epistemic, relational, and phronetic—none of which can be fundamentally sustained by an AI.

According to Pellegrino [29], a moral agent must understand moral norms, act consciously, and assume responsibility. Artificial intelligence systems do not meet any of these requirements. Even though artificial intelligence can optimise clinical data, it inherently lacks empathy and a ‘caring touch’ [33,34]. Although large language models (LLMs) can process ethical knowledge and generate the most likely solutions based on training data, such operations merely mimic rational thinking. Artificial intelligence lacks the consciousness required for moral agency and conscious engagement with what matters [35]. Furthermore, artificial intelligence lacks moral sensitivity, i.e., the uncodified, practical human ability to recognise morally significant aspects of a situation [36]. This deficit is not what larger datasets or more sophisticated architectures can remedy, since the knowledge in clinical judgement is more contextual than statistical. The missing point in this expectation is that moral responsibility—which requires full self-conscious awareness and the ability to comprehend what is right and wrong—is what makes an entity a moral agent [37]. Therefore, even ethically preprogrammed AI tools cannot be regarded as moral agents because they lack the three types of responsibility.

Epistemic responsibility. The clinician is responsible not only for what he or she knows, but for how that knowledge is applied to a specific individual. With AI, the question is no longer whether a prediction corresponds to reality, but what probability attaches to the predicted event. AI tools can produce an accurate prediction but not a moral justification of why a specific alternative should or should not be applied to a specific patient. Clinicians are presently better positioned to distinguish what is statistically probable from what is clinically right [38,39,40]. This emphasis on clinical rightness rather than statistical probability is not a denial of AI’s diagnostic accuracy. Where evidence establishes that a tool reduces error for a given clinical task, declining to use it without justification would be a failure of professional responsibility, not an expression of it. But accuracy and rightness are not the same property. As Kempt and Nagel argue, even a highly accurate AI ‘second opinion’ does not settle how its output should be integrated into a responsible clinical judgement. A ‘second opinion’ shifts rather than removes the deliberative burden, particularly where the system and the clinician disagree [41]. This deliberative burden is far from trivial: Rosenbacke et al. demonstrated empirically that when AI confirms a clinician’s initial assessment, clinicians tend to accept this agreement uncritically, producing ‘false confirmation’ errors in an estimated 5% to 30% of clinical decisions, depending on the accuracy of both the clinician and the system [42]. Notably, even explainable AI (XAI), which discloses the reasoning behind its output, does not meaningfully reduce this tendency, suggesting that confirmation by an algorithm can reinforce rather than challenge cognitive biases such as confirmation bias. In addition, a tool may improve accuracy across a population while still requiring phronetic interpretations of whether, and how, its output applies to this patient. The obligation to use accurate instruments and the obligation to interpret their outputs are complementary, not competing. Therefore, AI cannot be considered epistemically superior to human clinical reasoning [43].

Relational responsibility. Traditional in-person medical encounters, in contrast to digital or automated environments, consist of physical, personal contact, verbal explanations, and nonverbal emotional signals that are necessary to build trust [44]. Even if an artificial intelligence tool were to meet the epistemological requirements of clinical reasoning, it would still lack the ability to respond to the patient as a suffering human being. Relational responsibility, in a Levinasian sense, requires a direct response to the call of another person’s suffering [45,46,47]. An algorithm processes biological and behavioural signals but cannot receive or perceive suffering as a call that demands an empathetic response. Delegating this responsibility to artificial intelligence could be viewed as negligence that violates human dignity, since the patient has no one to entrust with his or her vulnerability. Therefore, discussions about AI ‘autonomy’ often miss the fact that, although these systems process data adaptively, they lack experiential or relational grounding [48]. Gordon and Gunkel [49] rejected the exclusion of AI from moral agency on the basis of intrinsic properties such as free will and propose a relational account of moral status. However, the relation that grounds moral agency in clinical practice is the face-to-face encounter, one into which AI cannot enter. The face-to-face relationship means, following Levinas’s concept, that a person exposes their inner self, their suffering, their vulnerability: “The skin of the face is that which stays most naked, most destitute. It is the most naked, though with a decent nudity. It is the most destitute also: there is an essential poverty in the face” [47] (p. 86). A person, when confronted with the suffering of another, or in Levinas’s words, with the face of the other, must assume an ethical obligation. In other words, a person cannot say no in the face of a suffering person who is asking for help. Taking responsibility for the other and preserving their dignity are connected to one’s own humanity [47].

Phronetic responsibility. Clinical responsibility requires practical wisdom, i.e., the capacity to act rightly in a particular case. AI cannot be regarded as a moral agent because it lacks the tacit knowledge [50] that the Aristotelian tradition calls phronesis: the application of general principles to a unique situation. Even where robotic AI tools exceed human performance in matters of techne, they do not act contextually. AI cannot recognise when a standard protocol should be suspended to safeguard a patient’s dignity. This requires the clinician to be able to interpret clinical findings in the context of the patient’s existential condition. This is the work of phronesis or, following Vallor’s [19] concept, ‘technomoral wisdom’. This wisdom is a ‘general condition’ of moral expertise that integrates and cultivates all the moral virtues necessary to respond to ethical challenges arising within the opaque and unpredictable technosocial reality of healthcare. Such wisdom cannot be delegated to algorithms. It is essential that the clinician acts as a moral interpreter—a team leader who considers both the values of team members and patients [51]. In this role, the moral interpreter carefully examines and interprets algorithmic system outputs while maintaining ethical vigilance. This requires moral courage—“the willingness to question, resist, and reframe decisions that may be procedurally justified but morally deficient” [52].

These three responsibilities clarify why clinical moral agency cannot be transferred to a system. Of course, it would be naïve to contrast a flawless human clinician with a flawed machine. Human clinical reasoning is itself heuristic, subject to cognitive bias, fatigue, and the pull of routine, and it has long been shaped by protocols and consultative tools. Clinical cognition operates under intrinsic constraints, including limited working memory capacity, temporal decay, and a reliance on intuitive reasoning that render clinicians vulnerable to anchoring, availability bias, and premature closure, particularly in high-pressure environments [53]. It might therefore be objected that, since clinicians err in any case, little is lost by deferring to a statistically accurate system. The problem is that the cognitive limitations that make clinicians imperfect do not disappear when AI is introduced. They re-emerge as a disposition to over-rely on outputs that are not fully transparent. Clinicians who interact with skewed algorithmic recommendations can absorb those distortions and reproduce them in later judgements made without algorithms [54]. Consequently, two trajectories may evolve: a critically engaged, morally responsible clinician can use AI to augment their reasoning and reach better decisions, whereas a clinician who does not engage critically risks de-skilling and may blindly adopt algorithmic outputs [55]. The ethical concern, accordingly, is not the existence of decision support, which has informed clinical judgement for decades, but the displacement of deliberative responsibility from an accountable agent to a system that cannot bear it. Even an ethically trained system cannot apply a moral fix by itself, although it could augment the ethical reasoning skills of a critically thinking clinician. This is exactly why the deliberative space must remain open rather than assumed: only within a protected space of judgement can clinicians continue to feel, and to assume, full responsibility for the decision.

## 5. Anthropomorphism, Algorithmic Authority, and Erosion of Trust

### 5.1. Anthropomorphism, Simulated Trust, and the Responsibility Gap

Despite the fact that AI systems of any kind do not possess the characteristics of a moral agent, the design of such systems can lead users into treating them as moral subjects. Clinical chatbots, for instance, simulate attentiveness and responsiveness in ways that their users or, in the case of clinical settings, patients might regard as more empathic than interactions with human clinicians (e.g., Hippocratic AI). This simulation of communication by artificial intelligence systems leads to anthropomorphism: users begin to attribute moral characteristics and even moral responsibility to systems that have none of these distinctly human traits [56,57,58]. Such attribution can create the illusion of a trusting relationship with an AI system, which poses significant risks in a clinical setting. Trust in general means placing something valuable in another person’s care in a vulnerable situation [59]. A clinician in traditional clinical settings is expected to honour ‘fidelity to trust’ [60]. However, in the case of AI, it is paradoxical for a patient to entrust his or her vulnerability to technology that has neither free will nor obligations.

The belief that we can expect help even in the worst situations is a critical element of trust. We put our trust in professionals who have the knowledge and skills to help. However, when a patient humanises AI and puts his or her trust in it, the real ‘saving hand’ does not exist. At the same time, the majority of clinicians and patients believe that clinicians should remain accountable for the errors of AI [9,61]. In clinical settings, this means that responsibility will depend on the clinician’s ability to recognise those errors and further ‘recklessness in acting’ [62]. A clinician’s ability to recognise errors, in turn, struggles with the ‘responsibility gap’, when the clinician is no longer able to predict the behaviour of the AI systems and therefore can no longer bear moral responsibility and liability for it [63].

Some experts, like Asadollahi [20], call this ‘responsibility choreography’. We agree with such a term for a situation where clinicians still retain all the accountability and culpability but have lost the actual power (agency) to make responsible and ethical medical decisions. They are expected to be responsible for the outcome but are not the ones allowed to decide it. We believe this is not just a technical issue—this is a crisis for the medical profession.

The responsibility gap might appear less novel than we have suggested. Distributed responsibility is the normal condition of contemporary clinical practice, where decisions routinely emerge from the coordinated contributions of multidisciplinary teams, consultative networks, and institutional protocols [40,64]. In this sense, it might be objected that AI introduces nothing categorically new, since accountability has long been shared across multiple actors and layers of expertise. However, the distinctive difficulty with AI is not distribution as such, but its opacity. Traditional distributed accountability still presupposes that each contributing actor can, in principle, give an account of their contribution, and that such justification can be examined, questioned, and overridden by others within the network. The responsibility gap arises when a central determinant of the decision can no longer be reconstructed by any of the responsible parties. The clinician is then expected to hold accountability for an outcome without commensurate insight into, or control over, the system that shaped it. As Bleher and Braun argued, the diffusion of responsibility in AI-driven decision support is best addressed not by locating a single liable agent, but through discursive and deliberative arrangements that keep responsibility attributable [64].

This ‘responsibility gap’ is also a conceptual issue that causes real difficulties. The problem is that AI can make mistakes. For example, some AI tools are trained only on certain groups of people, which means that such tools will not work well when the patient population is highly diverse [65,66]. In addition, an algorithm might work in one hospital but fail in another because every place uses the technology differently or simply because of the difference in the level of competence between AI users [67]. Some experts say we should keep checking the AI throughout its life cycle to fix this [68,69]. However, this does not help the clinician during that particular moment. A clinician who is standing there with a patient cannot look inside the ‘black box’ to see why the AI gave a certain answer. Instead, the system just expects the clinician to stay alert and double-check the machine’s calculations against his or her own logic.

### 5.2. Algorithmic Authority and the Erosion of Autonomy

Obviously, vigilance is the quality clinicians are expected to have. They are meant to notice all the small bits of relevant information and their mismatches. In real-life clinical settings, however, clinicians often operate under time and bureaucratic pressure. Some studies suggest that under time pressure, clinicians tend to succumb to growing but not yet definitive concerns regarding ‘automation bias’ and accept the results generated by artificial intelligence without properly analysing them [70,71]. ‘Confirmation bias’ lies on top of automation bias, and clinicians often accept only the pieces of evidence that confirm the AI solution and ignore the contradicting ones [72]. In the long-term perspective, there is a documented risk of skill decay, whereby overreliance on AI may erode the very competencies clinicians need to recognise AI errors [73,74,75]. This threat is especially pronounced for early-career clinicians, who may fail to acquire foundational clinical reasoning skills in the first place—a phenomenon termed never-skilling [74].

As a result, the quality of the clinician–patient relationship suffers when an ‘algorithmic authority’ takes over human judgement [76]. Therefore, the clinicians’ perceived competence and commitment to patient welfare are needed to close the ‘reliability gap’, in which neither the clinician nor the patient can truly verify the work of the algorithm [77].

Otherwise, when the clinician loses autonomy, the patient loses it as well. We are seeing a new kind of ‘the computer knows best’ attitude, which has been called transitive paternalism [78,79]. AI prioritises data points over the human side of medicine, pushing the patient’s personal values to the side [80,81].

The current situation in the healthcare sector raises a complex ethical question: if statistical data show that artificial intelligence is more accurate than a clinician, are we morally obligated to use it? Pellegrino’s framework [29] would resist a simple affirmative. The obligation is real where accuracy is established, but it is an obligation to use the tool, not to defer to it. True treatment requires ‘the right action’, one that integrates the patient’s values with the specific clinical context. Such integration can only be performed by a human being as a moral agent [82]. Greater statistical accuracy therefore grounds a duty to consult the tool, but not a duty to let its output stand in for the clinician’s deliberative judgement. Clinical accuracy is important, yet this does not mean that it can replace the entire decision-making process.

## 6. From Regulatory Frameworks to Clinical Virtue: Meaningful Human Control

### 6.1. The International Regulatory Framework

Compliance with regulatory requirements alone cannot mitigate the complicated issues arising in healthcare, especially the ethical ones. To understand this, it is worth examining what EU Regulation 2024/1689 [83], better known as the AI Act, does and does not require.

The AI Act distributes responsibility across distinct actors, and it is important to be precise about who bears what. Providers (developers) carry the principal burden for the safety of the system as a product—data governance, accuracy, robustness, technical documentation, and the design of human-oversight capability (Arts. 9–15). Deployers—in healthcare, typically the institution rather than the individual clinician—must use the system within the provider’s instructions and, crucially, assign human oversight to a natural person with the necessary competence, training, and authority (Art. 26(2)). The clinician enters this scheme not as a directly regulated actor but as the natural person to whom oversight is assigned. The Act therefore locates the clinician at its thinnest point: accountable for exercising oversight, yet dependent on a system designed by the provider, deployed by the institution, and operated within instructions that neither the clinician nor often the institution can independently verify. This is the regulatory form of the responsibility gap—the clinician holds accountability for outcomes without commensurate control over the determinants of the system’s behaviour.

The AI Act’s Article 14 requires that high-risk AI systems “be designed and developed <…> so that they can be effectively overseen by natural persons” during their use. Under Article 14(4), such oversight must allow a human to “properly understand the relevant capacities and limitations” of the system, to “remain aware of the possible tendency of automatically relying or over-relying on the output”, to “correctly interpret the <…> output”, to “decide not to use the system or to disregard, override, or reverse the output”, and to “intervene in the operation… or interrupt the system”.

These provisions directly address some of our concerns and require higher control over AI systems than nominal oversight. However, we argue that several gaps remain. First, the provisions of the AI Act are procedural in nature and do not address the question of what overriding AI outcomes should preserve, namely the integration of evidence, context, and patient values into the decision. Second, the Act sets the oversight mechanism primarily as a design feature of the AI systems, not as a structural element of clinical practice. An AI system may be designed so as to allow overriding its outputs; however, the clinical setting may penalise the clinician for it, as it would require more institutional (time, human resources) and economic (additional tests) resources. Third, the Act treats the overseeing human as a ‘natural person’, not as a moral agent within a professional tradition who possesses intrinsic values. Pellegrino’s understanding of the ‘act of profession’ is not captured by the concept of a natural-person operator.

It should be noted that scholars’ opinions on the evaluation of the effect of the AI Act differ. The AI Act sets an umbrella-like set of rules, and other EU- and national-level legal instruments cover healthcare-sector-specific issues [84]. Others note that significant gaps remain in questions of responsibility and oversight [85]. Despite the obvious progress, guidelines tailored to the requirements of the clinical setting still need to be developed in order to ensure a safe and trustworthy use of AI [86,87].

The AI Act tries to solve issues related to AI integration in healthcare technically, yet it does not address the core ethical problem of the structural erosion of the clinician’s moral agency. Based on the analysis above, we consider questions of responsibility and oversight worth understanding beyond the limits of formal requirements.

The WHO’s guidance [88] on the ‘Ethics and Governance of Artificial Intelligence for Health’ likewise sets the aforementioned requirements as key governance elements, but also expands this framework by noting ethical requirements for protecting patient autonomy, building trust, preventing harm, and ensuring fairness.

Both documents regulate procedural compliance and mitigate technical harm. However, they do not address the question of how clinical judgement and human dignity should be protected under AI pressure. Neither the EU Act nor the WHO guidance prevents the erosion of trust or the ‘statistical optimisation’ of moral duty. ‘Human oversight’, unfortunately, mainly reflects only technical control functions.

### 6.2. The ‘Meaningful Human Control’

We argue that another concept needs to be introduced. ‘Meaningful Human Control’ (MHC), we believe, is a better option, because it is more oriented towards the aspects of enabling, enacting, and evaluating control [89].

AI systems, as of today, are not yet ‘ethical rational agents’ able to recognise moral rules and operate them [90]. Therefore, in the decision-making process, humans should be in the driver’s seat, while AI systems should remain traceable to them [91]. In this regard, MHC should represent the idea that humans must keep real and effective oversight over AI systems in order to keep decisions, responsibilities, and societal values as being shaped by humans [92]. Understanding such a purpose of MHC is essential because it shapes the institutional design of healthcare settings [93].

We do not diminish the importance of the role of the EU AI Act—it is a crucial regulation oriented towards safeguarding the population from the risks arising from advanced technology. We state that the EU AI Act is a necessary but not-sufficient condition to ensure that advanced technology works well to meet the best interest of the patient. The difference between the Act and the framework we propose is ultimately a difference between two questions addressed to two different actors. The Act asks whether a system is safe to operate, and it assigns that question mainly to providers and to deployers, who deploy the system and assign a competent overseer.

The question of system safety is answered before and around the clinical encounter. Meaningful Human Control asks a different question—whether the system’s use is appropriate for this patient. This question can be answered only inside the encounter. Under the Act the clinician is the assigned overseer of a tool, but in the encounter the clinician is something the Act cannot see: a moral interpreter who, through phronesis, navigates between the tool and the patient toward the patient’s good.

The AI system uses the mathematical language of probabilities, classifications, and population-level patterns, while the patient presents a singular life, a biography, a set of values, a suffering. The clinician’s task is to interpret between them—to read the tool’s output and translate the patient’s situation into terms against which the output’s fit can be judged. This is the work of a moral interpreter, and it can be performed through phronesis as a capacity to discern the right action in the particular case, where AI-induced knowledge cannot determine what should be done for this person. The interpreter’s place between technology and patient cannot be filled by any system, because it is defined by standing in moral relation to the patient, which cannot automatically be grasped. The following table summarises this distinction (Table 1).

MHC, so understood, functions as a moral precondition for virtuous clinical practice. It exists to preserve the internal morality of medicine in the sense Pellegrino [12] defined. However, the two ideas need to be kept distinct here. The virtue is what clinicians cultivate themselves. Pellegrino [94] called it ‘a habitual moral disposition to make right choices in complex clinical circumstances’, alongside the virtues of fidelity to trust and intellectual honesty. MHC, by contrast, is the institutional condition that allows this virtue to be exercised. Even the most virtuous clinician cannot practise medicine according to its internal morality if the surrounding structures do not permit it. Our concern in this section is primarily with securing those structures.

A dignity-oriented reading of MHC yields three interdependent obligations. Clinicians must retain epistemic, moral, and relational authority. These together form the baseline for dignity-preserving healthcare, and they correspond directly to the three responsibilities we examined earlier in connection with moral agency. Under epistemic control, it is necessary to ensure that clinicians understand the limitations, uncertainties, and biases of AI systems in order to counter automation bias and protect the relational autonomy of clinicians. Moral control means that clinicians keep the freedom to engage with morally sensitive issues without algorithmic pressure, which in turn requires acknowledging that AI cannot detect moral context and cannot perceive what is ethically at stake in a given clinical situation; this parallels phronetic responsibility. Relational control means that clinical interactions preserve the interpersonal conditions constitutive of dignity and allow patients to participate meaningfully in decisions about their care; this parallels relational responsibility.

### 6.3. Normative Requirements for MHC

To ensure that MHC can function as an effective and ethically grounded safeguard in AI-supported healthcare, we propose five normative requirements. These are not five independent demands but five conditions for a single role—the clinician as moral interpreter—each protecting a distinct object: decision, deliberative space, epistemic access, the patient’s participation, and the selection of tools. We do not claim that they exhaust every requirement that might emerge as deployment patterns change, but we regard them as core conditions in whose absence Meaningful Human Control cannot be sustained. It is worth noting what sets these requirements apart from ordinary professional autonomy. AI adds scale, opacity, and institutional-statistical authority to clinical recommendations in ways that can displace deliberation rather than inform it. The requirements provided below take these characteristics into account.

Primacy of Clinical Judgement. If Meaningful Human Control is to exist, clinical judgement cannot be made subordinate to algorithmic output. Clinicians need the authority to override or set aside AI recommendations whenever those recommendations conflict with the patient’s values, the clinical context, or the clinician’s own professional assessment. A clinician who cannot decline an AI recommendation cannot sensibly be held accountable for its consequences. A patient whose care is dictated by an unchallengeable algorithm has been cut off from the relational and interpretive dimensions that uphold personal dignity in medicine. Protecting the primacy of clinical judgement therefore protects both the clinician’s moral agency and the patient’s dignity. This requirement protects the decision itself—the interpreter’s authority to determine what should be done rather than merely to ratify what the system proposes.

In image analysis, primacy of clinical judgement means that a flagged or unflagged finding does not by itself determine the diagnosis. The radiologist should retain authority to read the image against the clinical question and the patient’s history. In CDSS, it means that a risk score or a recommended treatment pathway enters the decision-making process as evidence, not as a verdict, and that the clinician may select a different pathway when the patient’s values or context dictate it. In ambient and generative tools, it means that the structured note or summary produced by the system is reviewed and corrected by the clinician, who remains the author of the clinical record rather than its editor. In each case, the requirement protects the same capacity—the clinician’s authority to determine what should be done.

Prohibition of Forced Automation. When clinicians are compelled to follow algorithmic pathways, they become operators of a technical system rather than independent moral agents. This danger is partially addressed by the AI Act, where Article 14(4)(b) requires natural persons to remain aware of the tendency to over-rely on outputs. Awareness, however, is not protection. Automation bias is not overcome by being warned of it when the workflow creates an acceptance path of least resistance. System designs that enforce such compulsion also heighten the danger that patients will be reduced from persons to data points, undermining the interpersonal respect that clinical practice should embody. This ethical requirement transcends the procedural safeguards currently provided by the WHO and the EU. Hospital governance, professional accreditation bodies, and departmental protocols each have a part to play in ensuring that forced automation does not become entrenched in clinical workflows. This requirement protects the deliberative space: departure from the output should be equally possible as acceptance in order not to push the clinician from interpreter to transmitter, who passes the output along to the patient rather than verifying whether it aligns with the patient’s needs and interests.

The prohibition of forced automation is most acute where throughput and time pressure are greatest. In image analysis, workflow designs that auto-advance accepted findings and require justification only for disagreement can determine acceptance. In CDSS, where a system-preferred pathway may be highlighted as a default, it should not penalise clinicians for justified, value-based departure from the recommendation. In ambient tools, it is important to ensure that clinicians are not compelled to adopt system-generated content without review simply because review is slower than acceptance.

Traceability and Explainability. Decisions supported by AI ought to be traceable and, as far as possible, explainable. The aforementioned WHO and EU documents already require transparency, traceability, and explainability, but they frame these mainly as governance mechanisms. We argue that clinicians must understand enough about how an AI system generates its outputs to judge whether those outputs are reliable and where their limits lie. Traceability should make it possible to reconstruct decisions by retracing key points in the AI’s reasoning process. Explainability should give clinicians enough information to integrate AI outputs into their own deliberation. This requirement protects the clinician’s ability to grasp the output’s assumptions and blind spots well enough to recognise where it does not fit this patient.

Traceability and explainability should be calibrated to what each AI system can offer and what the clinician needs. For image-analysis tools, for which deep-learning architectures are often least transparent, traceability means at minimum knowing the system’s validated performance characteristics, its known failure modes, and the sociodemographics of the populations on which it was trained, so the clinician can judge when its output is reliable for this patient. For CDSS, it means knowing which variables are estimated, on what assumptions the output is based (for instance, risk reduction presupposes full adherence), and whether the output is population-level or individualised. For generative tools, it means knowing what the system may have omitted or reformulated, since what is left out of a structured summary can matter as much as what is included. Wherever possible, explainability should include an indication of the model’s level of confidence in its conclusion.

Transparency towards Patients. Patients should know when AI is being used in their diagnosis or treatment. They should be told the system’s purpose, potential benefits, risks, and limitations. Such transparency lets patients ask questions, express preferences, and understand how their treatment is proceeding. Dignity-preserving communication of this kind reinforces trust in both the individual clinician and the healthcare system as a whole. WHO and EU frameworks treat transparency as a procedural requirement and do not ground it in dignity or recognise it as a moral practice foundational to trust. Article 14 itself does not directly impose a transparency duty on the overseeing clinician. Such duties are listed elsewhere in the Act (Arts. 13, 52) and in the Medical Device Regulation (Annex I) [95]. Transparency translated by the clinician is crucial to protect the patient’s right to participate in shared decision-making.

In image analysis, patients should be informed when AI has contributed to a diagnostic reading and to what extent. In CDSS, patients should understand that the recommended treatment pathway has been developed with the assistance of AI, be informed of the available alternative options, and know that the final decision depends on their personal values. In cases of generative AI, patients should know whether their consultation is being processed by AI and how that record will be used. In every case, transparency is not merely a disclosure requirement, but a condition of the dialogue through which shared decisions are made.

Retaining Clinical Authority. The patient’s right to give or withhold informed consent for a particular treatment should not be stretched to include limiting the clinician’s choice of diagnostic or decision-support tools needed to deliver safe and responsible care. Selecting such tools, including AI systems, falls within the clinician’s professional responsibility. MHC, therefore, requires that clinicians keep authority over the methods and technologies through which they discharge their moral and professional duties. A clarification is needed here. This requirement concerns clinical judgement authority over tools used in a specific case, not budgetary authority over institutional resources. Institutions legitimately decide which tools are available, while clinicians decide which available tools to use in a given situation. This distinction separates the professional autonomy we are defending from any broader guild claim over healthcare resources. This requirement protects the selection of tools: the clinician must remain free to decide which tools enter the encounter.

The following table illustrates the differences in the understanding of these requirements under ‘Human oversight’, as introduced in Art. 14 of the EU AI Act, and our proposed framework of ‘Meaningful Human Control’ and its impact on epistemic, relational, and phronetic responsibilities, as well as the role of the patient within those frameworks (Table 2).

Where do these five requirements take effect? They span several levels of healthcare governance. Hospital governance bodies must ensure that institutional policies neither mandate forced automation nor undermine clinical authority. Professional societies and accreditation bodies should weave MHC principles into standards of practice and continuing education. Medical licencing authorities need to ensure that competence frameworks reflect the skills needed to meet responsible AI-mediated practice demands. Departmental protocols must translate these principles into concrete workflow arrangements that preserve deliberative space for clinical judgement.

**Figure 1:**
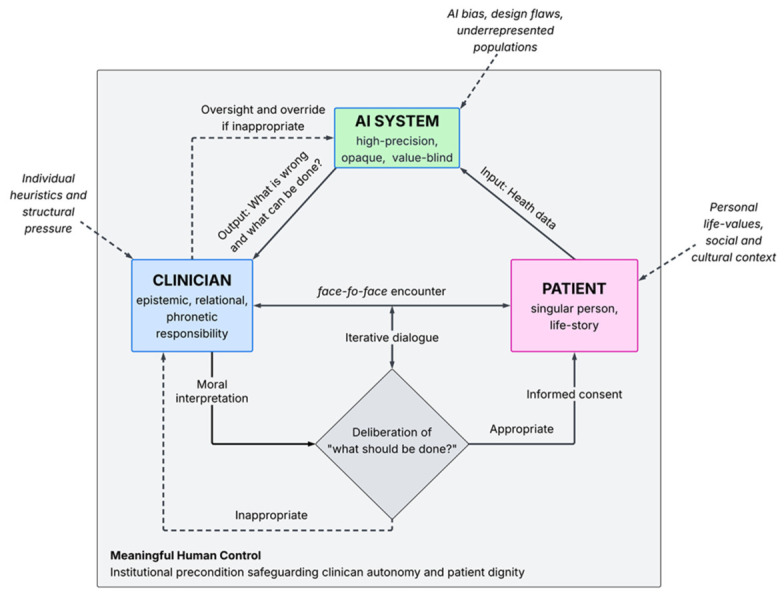
Clinical workflow under ‘Meaningful Human Control’.

Figure 1 brings these elements together. After a patient’s health data is passed through the AI system, which is high-precision but opaque and value-blind, the AI system generates output about what is wrong and what can be done. This output reaches the clinician who, by assuming epistemic, relational, and phronetic responsibility, exercises oversight of the AI output as required by the AI Act. Yet, the decisive work happens elsewhere. In the face-to-face encounter, the clinician hears the vulnerable patient as a singular person with a life story, life values, and a social and cultural context, and through moral interpretation reads the algorithmic output against this particular life. The deliberation space, ‘what should be done?’, is where the iterative dialogue between clinician and patient produces an informed choice. If the AI recommendation is not appropriate for the exact clinical case, the clinician overrides the AI outputs and, through iterative dialogue, seeks alternatives. If the decision is appropriate, it is implemented through the informed consent procedure. The whole process is enclosed by Meaningful Human Control as the institutional precondition that safeguards clinician autonomy and patient dignity.

### 6.4. Coming Back to Phronetic Responsibility

Retaining clinical authority means that the choice of which tools to use and how to weight their outputs remains a professional judgement rather than a patient entitlement or an imposed institutional mandate. A radiologist’s decision to consult or set aside an image-analysis tool, a family clinician’s decision to follow or depart from a CDSS pathway, a clinician’s decision to rely on or correct a summary—each falls within the clinician’s professional duties of care.

These five requirements span several levels of healthcare governance. Hospital governance bodies must ensure that institutional policies neither mandate forced automation nor undermine clinical authority. Professional societies and accreditation bodies should weave MHC principles into standards of practice and continuing education. Medical licencing authorities need to ensure that competence frameworks reflect the skills that responsible AI-mediated practice demands. Departmental protocols must translate these principles into concrete workflow arrangements that preserve deliberative space for clinical judgement.

Conflicts may arise in implementing these requirements, and they take different forms across modalities. In image analysis, explainability may conflict with the performance advantages of opaque deep-learning models. In CDSS, clinical autonomy may conflict with institutional requirements for efficiency or cost reduction, and the prohibition of forced automation may be hardest to sustain where a system-preferred pathway is highlighted as a default. In high-throughput settings such as radiology or intensive care, the pressure to accept system outputs by default is greatest. Transparency toward patients may sometimes increase anxiety. Our framework resolves these conflicts by prioritising the clinician’s phronetic responsibility—the capacity to act as a moral agent toward a specific patient—over technical optimisation. Where a requirement and an institutional or technical demand conflict, the test is whether the clinician’s ability to exercise moral agency is preserved. If it is not, the demand that undermines that agency must yield.

## 7. Conclusions

The ethical implementation of artificial intelligence in clinical settings requires more than human oversight over algorithmic outputs. A dialogue between the clinician and the patient as the sole moment that integrates scientific knowledge, the patient’s values, and situational context should remain the cornerstone of clinical decision-making. Artificial intelligence-based systems lack moral sensitivity and relational presence, and therefore they cannot safeguard the dignity that this dialogue is meant to protect. For that reason, they cannot presently be granted full membership in it. However, artificial intelligence progressively affects the processes of diagnostics and treatment. Under such pressure, defending the borders of clinical responsibility and trust becomes essential.

The analysis of dignity in the philosophical and clinical context, as conducted in this article, shows that patients depend on clinicians not only because of the technical skills the clinicians possess. Patients put their trust in clinicians due to their interpretive and relational engagement, which grants ethical validity to clinical decisions. If the boundaries are blurred, clinicians risk losing the authority they need to perform their professional duties. However, they will remain legally and morally responsible for results beyond their control.

The ground principles on which we believe AI-assisted healthcare should be built include the primacy of clinical judgement, the prohibition of forced automation, traceability and explainability, transparency towards patients, and retaining clinical authority. The understanding of these principles should go beyond seeing them as administrative protocols and technical requirements. First of all, they are meant to protect the conditions under which virtuous clinical practice can continue in an AI-assisted era. Each principle safeguards a specific capacity clinicians should be able to exercise: to align evidence with values, to resist algorithmic pressure and reconstruct its outputs, and to choose the proper tools and reveal them to the patient. Taken together, they aim to preserve what Pellegrino called the internal morality of medicine, in a form that sits comfortably with both technological progress and established medical ethics. Whether these five requirements will prove adequate to that task is a question that ongoing evaluation must answer as AI deployment evolves and new difficulties surface.

Artificial intelligence should not override clinicians’ capacities but rather support and enhance them. Patients’ dignity requires protection in the digital era. Responsibility for it lies on the shoulders of clinicians and manifests their moral duty towards their patients. We argue that Meaningful Human Control can be reached primarily through meaningful communication between the clinician and the patient. How this communication will be constructed, how clinicians and patients will react to it, and which points of it will be most vulnerable to algorithmic pressure—these are empirical questions that remain open. Qualitative and observational studies of AI-mediated clinical encounters could test and refine the normative framework we have developed here.

## Figures and Tables

**Table 1:** Comparison of the concepts of the EU AI Act regulation and Meaningful Human Control.

Focus	EU AI Act (Art. 14)	Meaningful Human Control
Question	Is the system safe to operate?	Is its use appropriate for this patient?
Answered by	Provider (safe design) and deployer/institution (safe deployment; competent overseer assigned).	The clinician, as moral interpreter, in the clinical encounter.
Locus	Before and around the encounter; about the system as a product.	Inside the encounter; about this patient’s good, values, and life.
Relation to the clinician	The clinician is the assigned overseer of a tool.	The clinician is the moral interpreter navigating, through phronesis, between tool and patient.
Guiding orientation	Procedural safety; necessary but not sufficient.	Clinical moral appropriateness; what safety cannot establish.

**Table 2:** Comparison of EU AI Act and Meaningful Human Control frameworks in AI-supported healthcare.

Dimension	Human Oversight (AI Act, Art. 14)	Meaningful Human Control
Core purpose	Procedural ability to intervene, monitor, or override AI outputs	Normative grounding ensuring moral agency, dignity, and context-sensitive judgement
Primacy of clinical judgement	Not required. Oversight frameworks do not mandate that clinical judgement takes precedence over AI outputs	Clinical judgement must remain primary. Clinicians must override AI when patient values, context, or moral reasoning require it
Traceability and explainability	Governance mechanism for accountability	Epistemic tool enabling clinicians to integrate AI into reasoning
Transparency	Procedural disclosure obligations	Transparency as a dignity-based moral practice enabling trust
Clinical authority	Not addressed; focuses on system oversight	Clinicians retain authority over diagnostic and decision-support tools
Patient relationship	Not central	Central: preserves relational, interpretive, dignity-based care
Epistemic responsibility	Clinician verifies whether inputs are correct and confirms outputs before communicating with patient	Clinician interprets AI through therapeutic relationship, contextual knowledge, and patient’s lived experience
Relational responsibility	Clinicians generally follow AI-supported protocols unless contraindicated	Clinicians integrate biomedical and biographical context, exercising practical wisdom to adapt or override AI plans
Phronetic responsibility	Interface allows overrides to meet regulatory requirements	Institutions must protect clinicians from audit pressure, metric penalties, or workflow constraints when deviating for justified reasons

## Data Availability

No new data were created or analysed in this study. Data sharing is not applicable to this article.
